# The state of the art: immune-mediated mechanisms of monoclonal antibodies in cancer therapy

**DOI:** 10.1038/sj.bjc.6605349

**Published:** 2009-10-06

**Authors:** J Griggs, K Zinkewich-Peotti

**Affiliations:** 1 UCB, 216 Bath Road, Slough, Berkshire SL1 4EN, UK

**Keywords:** antibody, Fc receptor, ADCC, fucose

## Abstract

A number of antibody products have now become accepted as effective anti-cancer therapies. Despite being mainly designed to act by inhibiting functional tumour antigens, there is increasing evidence that Fc-mediated engagement of the immune system is an important contributor to the efficacy of several of these therapies. The optimisation of this engagement offers the potential not only to augment efficacy against existing targets, but also to exploit non-functional tumour antigens. Antibodies that achieve efficacy wholly or predominantly through Fc-mediated mechanisms, represent rich opportunities for future therapeutics in oncology. This mini review summarises some of the key challenges, which need to be addressed to select the most effective molecules. These include the identification of optimal antibody characteristics and improvement of the drug discovery process, in particular, the relevance and predictive power of existing *in vitro* and *in vivo* screening methods. Advances in our understanding of tumour immunobiology and successful application of technologies designed to enhance immune system engagement will further aid this process.

The successful targeting of a range of cancer types with therapeutic antibodies, including bevacuzumab, trastuzumab and cetuximab, is driving substantial research into further novel biologics. There are currently six unconjugated antibodies approved in oncology by the FDA and in excess of 80 in various stages of clinical development (Reichert and Valge-Archer, 2007). In common with small-molecule approaches to targeted cancer therapy, antibody therapeutics have conventionally been designed to target mitogenic or pro-survival pathways aberrantly activated or differentially overexpressed in cancer cells as compared with that in normal cells. Well-characterised examples include members of the epidermal growth factor receptor (EGFR) family, which have been targeted both by small molecules, which effect receptor tyrosine kinase inhibition, and by antibodies, which inhibit signalling by binding to the extracellular domain of the receptor (e.g., cetuximab (Her-1) and trastuzumab (Her-2neu)).

Antibodies targeting such functional tumour antigens have principally been designed to achieve their effects through Fab-mediated mechanisms. By necessity, antibody engineering efforts have, therefore, focussed on optimising antibody–antigen interactions, for example, antibody affinity. A variety of technologies have been used to generate humanised or human antibodies to decrease immunogenicity (Almagro and Fransson, 2008).

Full-length monoclonal antibodies, including those targeting functional antigens, also have the capacity to act by additional mechanisms via their Fc domain, recruiting components of the host immune system to elicit cancer cell death. There are now compelling data indicating a significant contribution of such mechanisms to clinical efficacy. These data are accompanied by an increased understanding of the antibody biology involved in these processes. The potential to engage specific components of the immune system has become central to the rational design of novel antibody therapeutics to exploit Fc-mediated effects more fully. The focus of antibody drug discovery has, therefore, shifted somewhat in recent years, away from Fab generation, where many of the challenges have been met, and towards Fc engineering. Optimising the engagement of the immune system has the potential to augment the efficacy of antibodies directed against functional cancer cell antigens. Moreover, it presents the prospect of designing antibodies against non-functional tumour antigens to achieve efficacy wholly through Fc-mediated effects. This represents a unique opportunity to broaden substantially the range of antibody-based cancer therapeutics. Well-characterised functional tumour antigens (predominantly growth factor receptors) amenable to targeting by antibodies are scarce and many have now been intensively assessed either pre-clinically or clinically. In contrast, poorly characterised tumour antigens with unknown mitogenic or pro-survival functions, but with favourable tumour expression profiles, are comparatively abundant but have yet to be fully exploited. Previously described examples of tumour-associated antigens within this category include PSMA, 5T4, folate receptor-α, CEA and mesothelin (Reichert and Valge-Archer, 2007). Expression profiling strategies to identify antigens differentially expressed in tumours continue to expand this repertoire further. The successful application of new approaches and technologies to target these antigens constitutes a new challenge in the evolution of antibody therapeutics in oncology.

There are important differences in the drug discovery strategies that will be required to develop such antibodies as compared with those used to generate antibodies to inhibit functional tumour antigens. The development of antibody therapies against targets such as Her-1 and Her-2 was driven, at least in part, by their capacity to elicit direct and measurable effects on tumour cells induced by antibody binding both *in vitro* and in murine models (reviewed by Pegram and Slamon, 2000; Baselga, 2001). Studies using transgenic mouse models, inactive isotypes or depletion of specific immune cell populations have subsequently helped to dissect the contribution of Fc-mediated mechanisms to their efficacy (Clynes *et al*, 2000; Spiridon *et al*, 2004). However, the primary antiproliferative and other biological effects mediated by the Fab portion of the antibody could reasonably be expected to effect therapeutic gain and were the basis for the therapeutic hypothesis justifying entry into clinical trials. In contrast, the development of antibodies against novel targets, which may depend more, or solely, on Fc-mediated mechanisms of action, requires *in vitro* and *in vivo* screening techniques convincingly predictive for efficacy in humans. Our current screening cascades are confounded by immunological differences between rodents and humans, and our limited understanding of the parameters that influence how the interactions between target cells, antigen epitopes, antibodies, Fc receptors and effector cells lead to cell kill. Success will depend on addressing these current knowledge gaps and technological barriers, ultimately to understand the key antigen and antibody characteristics that are required to achieve the optimised and tailored recruitment of specific immune system components, and to unleash their therapeutic power.

## Fc-mediated mechanisms of immune system engagement

The mechanisms by which Fc–Fc-receptor interactions regulate immune responses to effect tumour cell death have been extensively reviewed elsewhere (Nimmerjahn and Ravetch, 2008). Briefly, therapeutic antibodies bound to tumour-cell surface antigens have the potential to elicit immune-mediated cell death either by engaging immune system effector cells or by activating the complement cascade.

Antibody-dependent cell cytotoxicity (ADCC) involves recruitment of immune effector cells (e.g., natural killer (NK) cells and macrophages) to kill cancer cells directly by a variety of means, including local release of granzymes and perforins. Similarly, antibody-dependent cell phagocytosis (ADCP) involves recruitment of phagocytic cells (e.g., macrophages) by virtue of their Fc-receptor expression, culminating in phagocytosis and death of the target cell. As discussed below, ADCP by cells with antigen presentation capacity also has the potential to engage the adaptive immune system. Understanding of resistance mechanisms to ADCC and ADCP beyond downregulation of the target is limited, although expression of Fc*γ*RIIb by melanoma cells has been proposed to decrease their susceptibility to ADCC (Cassard *et al*, 2008).

Human Fc*γ* receptors are expressed by a range of immune cell populations, including B cells, dendritic cells, macrophages, mast cells, NK cells and neutrophils (Desjarlais *et al*, 2007). They can be grouped according to their signalling transduction activities when engaged by their cognate Fc domain, as summarised in Table 1. Engagement of activatory Fc receptors results in phosphorylation of the cytoplasmic ITAM domain and subsequent signal transduction cascade, culminating in activation of cellular functions including ADCC and phagocytosis. In contrast, engagement of the inhibitory receptor Fc*γ*RIIb results in phosphorylation of its cytoplasmic ITIM domain and downregulation of the effector response. The physiological roles of Fc*γ*RIIb are varied and complex. Within the context of a negative regulator of immune activation, it plays a crucial role in regulating B-cell activity, modulating humoral tolerance and regulating plasma-cell survival. Of particular relevance to antibody therapeutics, multiple studies have also served to illustrate the importance of this receptor in regulating both innate and adaptive immune systems (reviewed by Nimmerjahn and Ravetch, 2008). The consequence of immune cell engagement by antibodies is tightly controlled by the balance of activatory and inhibitory Fc-receptor binding. The importance of this differential binding of antibodies is discussed further below.

There is significant variation in the affinities of IgG isotypes for individual Fc receptors, which is reflected by the capacity of active isotypes to recruit immune effector cells efficiently based on their Fc*γ* receptor expression profile. IgG1 and IgG3 are considered the principal active human isotypes based on their comparative affinity for activatory receptors. In the murine setting, IgG2a and IgG2b represent the most active functional isotypes (Dijstelbloem *et al*, 2001; Nimmerjahn *et al*, 2005a).

Complement-dependent cytotoxicity (CDC) results in the death of antibody-bound cells as a result of activation of the classical complement cascade. The process is initiated by the binding of c1q to the Fc portion of antigen-bound antibody and culminates in the production of a membrane attack complex in the target cell membrane, causing cell lysis. Resistance to CDC by membrane overexpression of complement regulatory proteins is evident in some tumour types (Yan *et al*, 2008).

## Data supporting the role of Fc-mediated mechanisms in antibody efficacy

An increasing body of data derived from both murine and clinical studies has helped to dissect the contribution of Fc-mediated activities to clinical efficacy.

Transgenic mouse models in which antibody–receptor engagement is modified or effector-cell activity is reduced have been extensively used for this purpose. For example, the efficacy of trastuzumab against breast cancer xenografts is dramatically inhibited in transgenic mice in which functional Fc receptors are not expressed, and is enhanced in models using mice deficient for the inhibitory Fc receptor Fc*γ*RIIB (Clynes *et al*, 2000). Furthermore, F(ab′)_2_ fragments of trastuzumab show poor *in vivo* efficacy against breast tumour xenografts as compared with full-length IgG, despite having similar *in vitro* antiproliferative and proapoptotic effects (Spiridon *et al*, 2004).

The most compelling data supporting the role of Fc-mediated mechanisms come from retrospective analyses of clinical data. These studies have generally focussed on attempting to correlate clinical response with Fc-receptor polymorphisms known to modulate antibody–receptor engagement. Specifically, Fc*γ*RIIIA 158V/V homozygosity confers approximately five-fold increase in affinity for IgG1 compared with the Fc*γ*RIIIA 158F/F genotype and NK cells isolated from donors with the Fc*γ*RIIIA 158V/V genotype show superior NK-mediated ADCC capacity *in vitro* (Dall'Ozzo *et al*, 2004). The clinical correlate of this observation was initially described in retrospective analyses of clinical responses to rituximab in which patients harbouring the Fc*γ*RIIIA 158V/V genotype showed superior response to rituximab in the treatment of non-Hodgkin's lymphoma compared with homozygous F/F or heterozygous F/V patients (Cartron *et al*, 2002). Further studies have consolidated these findings and, in addition, have demonstrated the importance of an additional polymorphism, Fc*γ*RIIA 131H/H (Weng and Levy, 2003), although the mechanism by which this polymorphism achieves superior engagement is not yet fully defined, as it does not show differential IgG1 affinity (Desjarlais *et al*, 2007). More recently, data relating to the prognostic significance of these polymorphisms in response to other antibodies, including trastuzumab (Musolino *et al*, 2008) and cetuximab (Bibeau *et al*, 2009), have been reported, which indicate similar trends. However, it should be noted that such data are not without controversy. Studies to date have used relatively small sample sizes and retrospective data analysis. Furthermore, apparently contradictory data describe the Fc*γ*RIIIA 158F/F genotype as being favourable for cetuximab response (Zhang *et al*, 2007). Prospective clinical trials with larger patient numbers are required to validate the role of Fc-mediated mechanisms. This will inform a variety of strategies aimed at maximising patient response, including genotyping patients to predict response and development of next-generation antibodies, which achieve efficacy irrespective of Fc-receptor polymorphism status. Such analysis may be confounded by other, independent factors that influence responses to targeting functional tumour antigens, for example, PI3-kinase mutation status in trastuzumab therapy (Junttila *et al*, 2009) and K-ras mutation status in cetuximab treatment (Bibeau *et al*, 2009).

## *In vitro* characterisation of antibody repertoires

Repertoires of antibodies against a specific candidate target can be generated by a variety of methods. Variable regions derived from such techniques can readily be grafted onto antibody framework constructs to generate repertoires of full-length antibodies of the desired isotype, and screened for properties including antigen affinity and specificity. Individual antibodies must also screened for ADCC activity, as repertoires can contain antibodies with significant differences in apparent ADCC potency, despite being specific for the same antigen and sharing properties of identical Fc and framework sequences, and similar binding and affinity profiles (Griggs *et al*, unpublished data). Such differences can be identified using a variety of robust human *in vitro* models for assessing CDC or ADCC activity. The lysis of target cells endogenously expressing the antigen of interest can readily be detected after co-incubation with human serum or purified human complement (CDC detection) or purified human PBMCs or NK cells (ADCC detection). Despite their utility in providing a means of confirming the potential for ADCC or CDC competence, such assays are not without their limitations. While they provide a system in which human target and effector cells and human format antibodies interact, little is known about the physiological relevance of the conditions used, particularly the ratio of effector and target cells employed, which is typically higher than might reasonably be expected to be achieved *in vivo*. The relevance of the effector cells used in ADCC assays *in vitro* also remains to be defined: NK cells are most commonly employed in this context but are not necessarily the most important effector-cell population for all tumours *in vivo*. Furthermore, as they do not express the inhibitory receptor Fc*γ*RIIb (CD32b), positive data from assays using NK effectors may be misleading as, for example, they would fail to account for the effects of inhibitory Fc-receptor interactions on other effector-cell populations. Significantly, the development of parallel *in vitro* ADCC assays using murine format antibodies and murine effector cells has proven challenging. This complicates the transition from *in vitro* to *in vivo* screens in the drug discovery process as human format antibodies identified *in vitro* may not elicit equivalent engagement of the murine immune system and therefore may not represent the most appropriate tools for target validation, nor necessarily constitute the lead candidates for further development. This is particularly problematic when attempting to validate poorly-characterised antigens and models.

One of the major determinants of the heterogeneity in potency seen within antibody repertoires is likely to involve the specific epitopes targeted. Surprisingly little attention has been given to the importance of epitope selection and the identification of optimal epitope characteristics, despite increasing evidence that targeting particular epitopes may have crucial implications for antibody efficacy. For example, the novel anti-CD20 antibody ofatumumab, currently in phase-III clinical trials and under review by the US FDA, is significantly more potent at inducing CDC than rituximab. Ofatumumab recognises a distinct epitope in close proximity to the plasma membrane, which is claimed to permit a more efficient localisation of complement components on the cell surface (Ruuls *et al*, 2008). Novel approaches have begun to address the potential of targeting multiple epitopes by combining several monoclonal antibodies against a single antigen. Such a ‘polymonoclonal’ therapy may be expected to achieve an additive therapeutic effect (Tolstrup *et al*, 2006), and this approach has progressed to clinical evaluation in several non-oncological disease areas. Encouraging preclinical data relating to polymonoclonal targeting of EGFR in oncology have recently been reported (Koefoed *et al*, 2009).

An additional consideration relating to the use of *in vitro* assays to select candidate antibodies for progression is that they fail to account for antibody properties which may influence tumour penetration *in vivo*. These properties remain to be fully defined, but are likely to include both physical and biochemical characteristics (Beckman *et al*, 2007).

## Uses and current limitations of murine models

The transitioning of candidate therapeutic antibodies into meaningful *in vivo* efficacy assays remains problematic, principally due to lack of equivalence between human and murine immune systems. Such issues appear to extend beyond species differences in Fc receptor repertoires and antibody isotype activities, as immune interactions may also differ between antigens.

Notwithstanding these difficulties, murine models can represent useful tools for target validation and proof-of-concept studies for novel tumour-specific antigens. Antitumour activity in ADCC- and/or CDC-competent models can be compared to that in models where Fc-mediated responses are impaired, to define the contribution of the immune system (Clynes *et al*, 2000). However, such studies do not necessarily lead to identification of the optimal human therapeutic candidate for progression into clinical trials. Significantly more sophisticated murine models may be required to achieve this aim and to avoid the risk of identifying antibodies with murine potency but sub-optimal capacity for human immune system interaction. Reconstituted SCID mice, in which human effector cells are engrafted into immunodeficient mice, have been employed as a means of artificially representing the human immune system *in vivo* (Shultz *et al*, 2007). Despite recent advances in the use of these models to evaluate ADCC activity (Ito *et al*, 2008), they have a range of limitations and do not represent a true reproduction of human effector-cell populations. As an alternative, transgenic mice expressing human Fc*γ*RIIIa human in the absence of murine Fc*γ*RI and Fc*γ*RIII are now available. The ultimate tool for the modelling of Fc-mediated ADCC by human effector cells may be a murine model fully transgenic for all human Fc*γ* receptors and devoid of endogenous murine Fc receptors.

Consideration must also be given to non-human primate immune system characteristics if such species are to be used for meaningful preclinical toxicology studies. Despite recent progress, our understanding of the equivalence of human and primate Fc-receptor engagement remains incomplete. The resolution of this issue will become increasingly important as antibodies with optimised immune activatory capacity are developed, particularly if target antigens show some degree of normal tissue expression.

## Technologies for enhancement of Fc effects

A variety of technologies have been developed aimed at optimising the antibody–immune system interaction to maximise Fc-mediated effects. The ultimate aim of such approaches is the development of antibodies with novel, tailored Fc regions able to elicit effects *via* one or more selected mechanisms of action that retain the favourable properties of specificity, affinity, low immunogenicity and acceptable pharmacokinetics. The main focus of several of these approaches has been to achieve increased Fc affinity for activatory Fc-receptors and/or decreased affinity for inhibitory receptors on immune effector cells to achieve enhanced ADCC. Within this context, strategies can be grouped into those which modify antibody glycosylation and those identifying key Fc amino-acid sequences for mutagenesis.

Antibody glycosylation has emerged as a major determinant of Fc-receptor affinity and this has been exploited by several technologies. In particular, antibodies with reduced fucosyl moieties show pronounced increase in potency in *in vitro* assays of ADCC using human effector cells (Shields *et al*, 2002). Crucially, it has been reported that low-fucose rituximab elicits enhanced ADCC irrespective of the Fc*γ*RIIIA 158V/F polymorphism (Niwa *et al*, 2004). Several afucosylated antibodies have now entered clinical trials (Table 2). The *N*-glycosylation of Fc domains with other moieties, including sialic acid, mannose and bisecting *N*-acetylglucosamine also has a dramatic effect on Fc–Fc-receptor interaction and is the subject of further technological approaches (Raju, 2008). It remains to be established whether these technologies augment ADCC to equivalent degrees in human and murine settings. Indeed, there are currently conflicting data on whether afucosylation of human antibodies increases binding to murine Fc*γ*RIV. Although it is possible that such interactions may be antibody-specific, this would be unexpected. Failure of afucosylation to alter interactions between human antibodies and murine Fc receptors would render the predictive utility of *in vivo* murine screens using such human antibodies questionable.

Fc mutational engineering presents an alternative approach to improving both ADCC and CDC potency. A variety of amino-acid residues that are critical for Fc–FcR interactions have been identified and modified to achieve increased binding to Fc*γ*RIIIa and/or decreased binding to Fc*γ*RIIb. Combinations of amino-acid substitutions have been applied to clinically validated antibodies, resulting in increased ADCC potency *in vitro* exceeding two orders of magnitude and increasing target-cell depletion *in vivo* (Lazar *et al*, 2006). Combining optimised mutational and glycosylation approaches does not appear to confer an additive advantage (Masuda *et al*, 2007). Amino-acid residues influencing C1q binding can also be manipulated to alter CDC activity, although the contribution of CDC to clinical efficacy is much less well described than that of ADCC. A variety of approaches have sought to combine enhanced ADCC and CDC activities, including Fc isotype domain shuffling (Natsume *et al*, 2008).

Ravetch and co-workers have defined an ‘A:I ratio’ as a means of ranking antibody Fc-mediated functional activity by calculating differential binding of antibodies to activatory and inhibitory receptors in biochemical assays. Striking data have been reported that correlate the A:I ratio of particular murine antibody isotypes against a common antigen with their capacity to achieve ADCC-mediated tumour inhibition *in vivo*. For example, the anti-gp75 antibody, TA99, is highly efficacious in an experimental melanoma metastasis model as a murine IgG2a format (high A:I), but demonstrates sequential loss of efficacy when used in IgG2b, IgG1 and IgG3 formats (decreasing A:I) (Nimmerjahn and Ravetch, 2005b). Such studies highlight the importance of isotype choice for eliciting Fc-mediated mechanisms. The extension of the A:I hypothesis into a differentiating screen for optimal drug candidates from within a panel of human antibodies with a common isotype is an enticing possibility.

There is renewed interest in IgEs for passive immunotherapy as IgEs have a natural affinity for Fc*ε*RI, which is 2–5 orders of magnitude greater than IgGs for their cognate receptors. IgEs can trigger ADCC and ADCP activity by eosinophils, mast cells and macrophages (Jensen-Jarolim *et al*, 2008).

Beyond antibody engineering techniques, Fc-mediated responses may also be augmented by increased activation, expansion and recruitment of effector cells. Approaches include administration of recombinant cytokines (e.g., interleukin-2 (IL-2), IFN-*α* and GM-CSF) or immunomodulatory drugs (e.g., thalidomide analogues). For example, preclinical observations (Lopes de Menezes *et al*, 2007; Reddy *et al*, 2007) have described a synergistic effect of IL-2 in combination with rituximab in both retuximab-sensitive and retuximab-insensitive non-Hodgkin lymphoma (NHL) xenograft models. However, a phase-II clinical study showed no benefit of rituximab IL-2 combination therapy in retuximab-refractory NHL (Khan *et al*, 2006).

## Identification of key effector-cell populations

The role of particular effector-cell populations in achieving ADCC *in vivo* remains controversial. While NK cells and macrophages share some common Fc-receptors, it understates the complexity of immunotherapeutic approaches to assume that a generic approach of increasing antibody affinity for activatory receptors will necessarily suffice for achieving optimal effector cell engagement. For example, NK cells are generally believed to be largely responsible for Fc-mediated efficacy against haematological tumours, yet reportedly show very poor penetration into solid tumours. However, this does not necessarily justify an emphasis on optimising macrophage engagement, as tumour-associated macrophages have the potential to exert pro-tumorigenic effects. Furthermore, data are emerging suggesting that antibody treatment may enhance tumour infiltration of NK cells. Thus, Fc-mediated antibody activities must be viewed in the context of the tumour microenvironment, which cannot be recapitulated *in vitro*. Further understanding of the role of specific effector-cell populations in particular tumour types is required to elucidate the optimal effector-cell engagement strategy for solid-tumour therapy, which may not prove the same for all tumour types. The complexity of the *in vivo* environment is well illustrated by work with anti-CD20 monoclonal antibodies where ADCC was shown in a murine model to be the main mechanism of action in most cellular compartments, but CDC drove B-cell depletion in marginal zones (Gong *et al*, 2005).

## Engaging the adaptive immune system

The engagement of the adaptive immune system against tumour antigens represents a long-standing goal in cancer therapy. The development of optimised passive immunotherapies may offer the potential to achieve such an adaptive immune switch. Enhancing the antibody-mediated recruitment of effector cells with antigen-presentation capacity, for example, dendritic cells, may lead to presentation of tumour antigens in an appropriate context to engage a T-cell-driven response. Preliminary data appear to confirm the potential of this approach and there is considerable interest in defining the conditions that need to be met to break immune tolerance to tumour antigens. Of particular interest are recent observations that describe the process of ‘cross-priming’ of professional antigen-presenting cells. Phagocytosis as a consequence of Fc-receptor engagement can result in preferential presentation of tumour-associated antigens by MHC class-I rather than MHC class-II, potentially activating CD8-positive effector T cells with tumour-antigen specificity (reviewed by Desjarlais *et al*, 2007). Intriguingly, the bias towards MHC class-II presentation appears to be driven by decreased inhibitory Fc-receptor binding. This raises the possibility that selecting antibodies with favourable A:I properties may achieve the dual benefit of enhanced ADCC and enhanced adaptive immune system engagement.

It should be noted that the clinical use of antibodies with putative Fc-mediated activity has not been without some toxicity in patients. In common with all methods that seek to increase Fc-mediated effects, the potential to increase toxicity should be considered, particularly in the context of autoimmune complications that may arise where normal tissue expression of the antigen occurs.

## Concluding remarks

Convincing evidence of the important role of Fc-mediated mechanisms of action in the clinical efficacy of antitumour antibodies now exists. Such evidence reveals not only the potential to augment the clinical efficacy observed to date against existing targets, but also the opportunity to exploit a range of other cancer targets with antibody therapeutics designed to achieve their effects predominantly through Fc-mediated mechanisms. There remain significant challenges within the drug discovery process to achieving this aim, most notably, the development of optimal *in vitro* and *in vivo* screening methods, an improved consideration of the importance of epitope selection and a better understanding of events at the tumour–immune system interface. Success in meeting these challenges will achieve a new generation of therapeutics and will represent an advance as important as previous technological achievements in antibody engineering that have enabled their successful clinical use.

## Figures and Tables

**Table 1:** Fc*γ*-receptors in human and mouse

		**Activatory**	**Relative**	**Structural signalling**	**Allelic**	**Putative functional murine**	**Principal effector-cell expression profile^a^**				
**Receptor**	**CD**	**inhibitory**	**affinity for IgG**	**components**	**variants**	**analogue**	**m*Φ***	**DC**	**N**	**B**	**NK**
*Human*											
Fc*γ*RI	CD64	Activatory	High^b^	ITAM (associated *γ*-chain)		Fc*γ*RI	•	•	•		
Fc*γ* RIIa	CD32a	Activatory	Low/Mod	ITAM (integral)	131H 131R	Fc*γ*RIV	•	•	•		
Fc*γ* RIIb	CD32b	Inhibitory	Low/Mod	ITIM (integral)	232I 232T	Fc*γ*RIIb	•	•	•	•	
Fc*γ* RIIc	CD32c	Activatory	Low/Mod	ITAM (integral)			•		•		•
Fc*γ* RIIIa	CD16a	Activatory	Low/Mod	ITAM (associated *γ*-chain)	158V 158F	Fc*γ*RIII	•	•			•
Fc*γ* RIIIb	CD16b	Activatory	Low/Mod	Non-signalling (gpi-linked)	NA1 NA2				•		
											
*Mouse*											
Fc*γ*RI		Activatory	High	ITAM (associated *γ*-chain)			•	•			
Fc*γ*RIIb		Inhibitory	Low/Mod	ITIM (integral)			•	•	•	•	
Fc*γ*RIII		Activatory	Low/Mod	ITAM (associated *γ*-chain)			•	•	•		•
Fc*γ*RIV		Activatory	Low/Mod	ITAM (associated *γ*-chain)			•	•	•		

Abbreviations: DC=dendritic cell; ITAM=immunoreceptor tyrosine-based activatory motif; ITIM=immunoreceptor tyrosine-based inhibitory motif; m*Φ*=macrophage; N=neutrophil, B=B cell; NK=natural killer cell.

a Fc-receptor expression is variable and can be modulated on certain cell populations, for example, by cytokine signalling and activation status. Data shown are intended to indicate expression on key effector-cell populations involved in Fc-mediated immune responses.

b Human and murine Fc*γ*RI have high affinity for monomeric IgG irrespective of antigen binding.

**Table 2:** Clinical-phase anticancer antibodies incorporating Fc modifications

**Ab name**	**Target**	**Technology**	**Clinical phase**	**Company**
XmAb2513	CD30	XmAb Fc^a^	Phase I	Xencor
MDX-1401	CD30	Potelligent^b^	Phase I	Medarex
BIW-8962	Ganglioside GM2 receptor	Potelligent	Phase I/II	Kyowa Hakko Kirin (Biowa)
Afutuzumab	CD20	GlycoMAb^c^	Phase II	Roche (Glycart)
R7160	EGFR	GlycoMAb	Phase I	Roche (Glycart)

Abbreviation: EGFR=epidermal growth factor receptor.

a Fc protein engineering to increase Fc receptor engagement.

b FUT8-knockout CHO cells producing non-fucosylated antibodies with increased Fc-receptor engagement.

c Genetically engineered cells producing antibodies bearing bisected non-fucosylated oligosaccharides with increased receptor engagement.
